# Clinical Outcomes of Transcranial and Endoscopic Endonasal Surgery for Craniopharyngiomas: A Single-Institution Experience

**DOI:** 10.3389/fonc.2022.755342

**Published:** 2022-02-10

**Authors:** Chuansheng Nie, Youfan Ye, Jingnan Wu, Hongyang Zhao, Xiaobing Jiang, Haijun Wang

**Affiliations:** ^1^ Department of Neurosurgery, Union Hospital, Tongji Medical College, Huazhong University of Science and Technology, Wuhan, China; ^2^ Department of Ophthalmology, Union Hospital, Tongji Medical College, Huazhong University of Science and Technology, Wuhan, China

**Keywords:** craniopharyngiomas, outcomes, transcranial surgery, endoscopic, surgery

## Abstract

**Objective:**

Craniopharyngioma has always been a challenge for the neurosurgeon, and there is no consensus on optimal treatment. The objective of this study was to compare surgical outcomes and complications between transcranial surgery (TCS) and endoscopic endonasal surgery (EES) of craniopharyngiomas.

**Methods:**

A retrospective review of patients who underwent craniopharyngioma resection at Wuhan Union Hospital between January 2010 and December 2019 was performed. A total of 273 patients were enrolled in this retrospective study. All patients were analyzed with surgical effects, endocrinologic outcomes, complications, and follow-up results.

**Results:**

A total of 185 patients underwent TCS and 88 underwent EES. There were no significant differences in patient demographic data, preoperative symptoms, and tumor characteristics between the two groups. The mean follow-up was 30.5 months (range 8–51 months). The EES group had a greater gross total resection (GTR) rate (89.8% EES vs. 77.3% TCS, p < 0.05) and lower rate of hypopituitarism (53.4% EES vs. 68.1% TCS, p < 0.05) and diabetes insipidus (DI) (51.1% EES vs. 72.4% TCS, p < 0.05). More postoperative cerebrospinal fluid (CSF) leaks occurred in the EES group (4.5% EES vs. 0% TCS, p < 0.05). More patients in the EES group with preoperative visual deficits experienced improvement after surgery (74.5% EES vs. 56.3% TCS, p < 0.05). There were statistical differences in the recurrence rates (12.5% EES vs. 23.8% TCS, p < 0.05) between the 2 groups.

**Conclusion:**

These data support the view that EES is a safe and effective minimally invasive surgery compared to TCS. Compared to TCS, EES has fewer surgical complications and a lower recurrence rate.

## Introduction

Craniopharyngioma is a rare benign tumor with a histologically low grade (WHO grade I) and mainly develops from remnants of the craniopharyngeal duct (1, 2). The annual incidence of craniopharyngioma is approximately 0.5–2.5 cases per million globally (3). Patients with craniopharyngiomas exhibit a bimodal age distribution of 5–14 years and 50–75 years (3). Although craniopharyngioma accounts for only 1.2%–4.6% of all intracranial tumors, it is considered to be the most common non-glial intracranial tumor in children, accounting for 10% of all brain tumors in children (4). The clinical manifestations of craniopharyngioma may occur due to compression or invasion by tumors, and the presenting symptoms may be different among children and adults. Symptoms of craniopharyngioma in children are often delayed, most of which are caused by the tumor growing to a considerable size. Children usually present with endocrine dysfunction, slowly progressive visual loss, and symptoms caused by increased intracranial pressure, while adults consistently have visual deficits (5, 6). The overall survival rates of childhood-onset craniopharyngiomas are 87%–95% at 20 years (2), and there are usually complications of hypothalamic–pituitary deficiencies, visual impairment, and neurologic dysfunction that led to a severe decline in long-term quality of life (1–3, 7). Craniopharyngioma is a surgical disease, and surgical management for craniopharyngiomas, especially in children, remains controversial (2, 8). The goal of treatment is permanent tumor control or cure without aggravating the symptoms. The aim of surgical resection is to achieve gross total resection (GTR) to reduce the risk of tumor residual and recurrence. Since the tumor is anatomically close to the optic nerve, third ventricle, and hypothalamus, it is critical to choose the appropriate approach to avoid serious postoperative complications like hypothalamic–pituitary dysfunction. Over the past decade, endoscopic endonasal surgery (EES) has been widely applied in the treatment of craniopharyngioma (9). Endoscopic surgery can provide a close high-definition view, which can clearly identify the anatomical structures, thus reducing intraoperative injuries. In contrast, traditional transcranial surgery (TCS) often requires retraction of brain and cranial nerves, especially the optic nerve, which often causes postoperative cerebral edema and cranial nerve impairment. Reports revealed that EES has significant advantages over TCS in intrasellar type of craniopharyngiomas (10, 11). However, for tumors located in the suprasellar region, there are relatively few studies directly comparing the surgical outcomes of EES and TCS. Both EES and TCS have their advantages, and there remains a lack of consensus on their benefits (12). In the current study, we retrospectively assessed outcomes of EES and TCS for suprasellar craniopharyngiomas.

## Methods

This retrospective study included all patients who underwent resection of craniopharyngiomas from January 2010 to December 2019 at Wuhan Union Hospital. All cases were pathologically confirmed as craniopharyngioma. Completely intrasellar craniopharyngiomas and recurrent cases were excluded. The medical records of all included patients were retrospectively reviewed. According to the records, patients were divided into the EES group and TCS group. Detailed patient records and follow-up reports were viewed to collect clinical data including symptoms; pathological, endocrinological, and ophthalmological assessments; and surgical outcomes. Ophthalmological assessments consisted of best corrected visual acuity and visual field examination. For both visual acuity and visual field, postoperative status was categorized as improved, stable, or deteriorated. To assess the visual acuity, the modified logMAR scale was used. To assess visual field deficits, an ordinal scale was used with the following scores: 6 indicates normal visual field; 5, slight constriction; 4, loss of a single quadrant; 3, loss of 2 quadrants; 2, loss of 3 quadrants; 1, severe constriction; and 0, blindness (13).

All the hypothalamic–pituitary axis hormones including plasma prolactin, thyroid function, growth hormone, luteinizing hormone (LH), follicle-stimulating hormone (FSH), estradiol, adrenocorticotropic hormone (ACTH), and plasma cortisol level were examined. All patients completed preoperative CT scans to detect the presence of calcifications. MRI was completed to identify detailed anatomy of the tumor and its relationship to the surrounding neurovascular structures. During the follow-up, MRI was performed at 1–2 days and 3–6 months after surgery. The tumor size was displayed as the largest diameter in all 3 dimensions (length, height, and width) on preoperative MRI. Tumor volume was calculated assuming a roughly spherical tumor configuration where tumor volume is in cubic centimeters (cm^3^) = (anteroposterior × craniocaudal × transverse)/2. The consistency of tumor was assessed based on MR images and intraoperative records.

**Figure 1:**
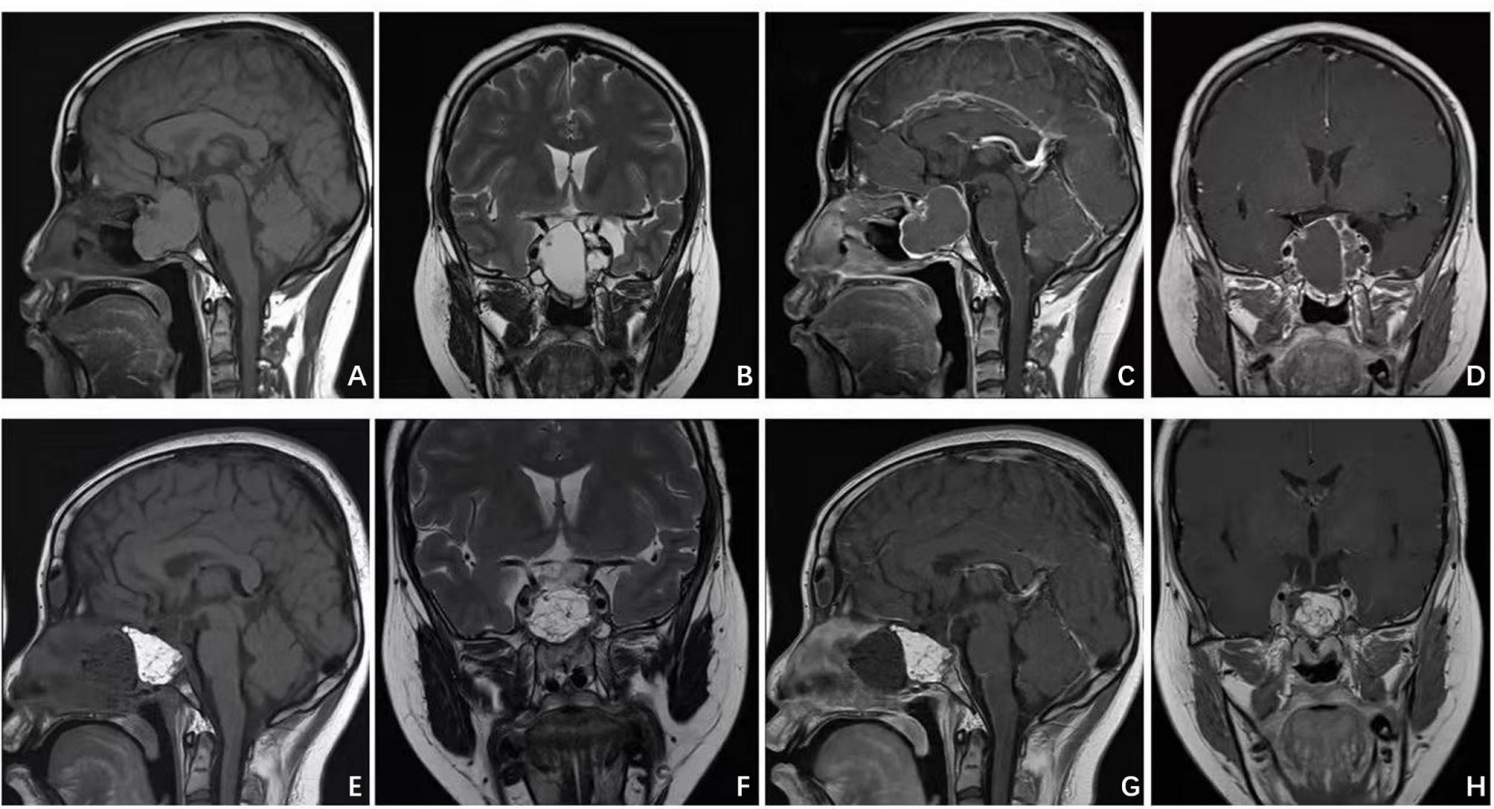
Patient presented with visual deficit, pituitary and elevated intracranial pressure syndromes. Preoperative MRI **(A–D)** illustrated a giant intra-suprasellar craniopharyngioma. Postoperative MRI **(E–H)** confirmed gross total resection.

**Figure 2:**
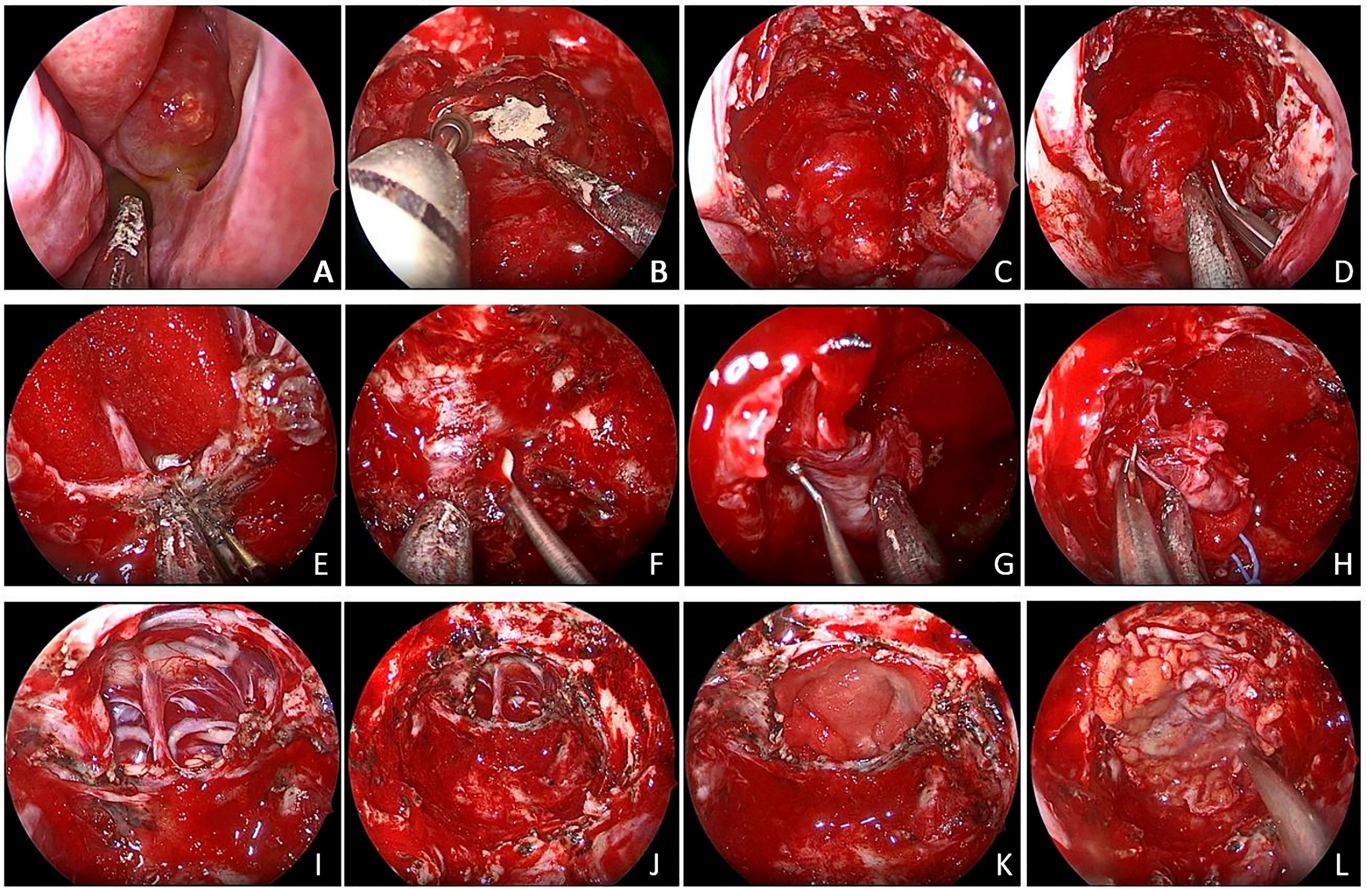
**(A)** The tumor broke through the sphenoid sinus and grew to the nasal cavity. **(B, C)** An extended transnasal approach was performed. **(D)** Decompression inside the tumor. **(E)** Removal of the saddle septum attached to the base of the tumor. **(F–H)** Remove the adhesion tissue between the tumor and the cavernous sinus and internal carotid artery. **(I)** Pituitary stalk was preserved. **(J–L)** Tumor was gross total removed.

We defined the extent of tumor resection as GTR and subtotal resection (STR). GTR was only assumed if there were no tumor or capsule remnants postoperatively on MRI examination. All surgeries were performed by senior experts in our department. The pterional approach, providing short distance to parasellar region, was our first choice in TCS for craniopharyngiomas. In some cases, subfrontal approach was adopted to achieve good visualization of optic nerve and chiasm as well as ipsilateral carotid artery. For tumors extending into the third ventricle, lamina terminalis or transcallosal approach was performed. Within the EES cases, the key rule was to protect the pituitary stalk and hypothalamus. To avoid cerebrospinal fluid (CSF) leaks, the overlay technique with a pedicled nasoseptal flap was applied to reconstruct the skull base. A case example is shown in **Figures 1**, **2**. Among patients with severe hydrocephalus, emergency EES or TCS combined with lateral ventricle drainage surgery was done.

### Statistical Analysis

Data were analyzed by SPSS 26.0. Descriptive statistics were used to analyze patient demographics. Continuous variables were described as means with SDs or medians as appropriate. Categorical variables were presented as frequencies or percentages. Group comparisons were assessed by the Student’s T-test, chi-square test. Differences with p < 0.05 were considered statistically significant.

## Results

### Clinical Characteristics

A total of 273 patients were enrolled in this study; 185 patients were assigned to the TCS group and 88 patients to the EES group.

The average age of the 273 patients in the study was 38.1 ± 12.9 years. The mean follow-up period was 30.5 months (8–51 months). The EES group included 41 (46.6%) males and 47 (53.4%) females, and the TCS group included 87 (47.0%) males and 98 (53.0%) females. In the EES and TCS groups, 51 (59%) of 88 patients and 118 (64%) of 185 patients were children, respectively, 37 (41%) and 67 (36.2%) were adults; headache occurred in 71 (80.7%) patients and 131 (70.8%) patients; symptoms of visual deficits presented in 47 (53.4%) and 87 (47.0%) patients; 9 (10.2%) patients and 15 (8.1%) patients presented with impaired cognition; 8 (9.1%) patients and 14 (7.6%) patients got obesity; hydrocephalus was noted in 11 (12.5%) patients and 28 (15.1%) patients; 33 (37.5%) patients and 75 (40.5%) patients had endocrine deficiencies before surgery. There were no significant statistical differences between these two groups with regard to the average age, sex, and preoperation symptoms. **Table 1** shows the demographic characteristics and clinical features in each group.

**Table 1:** Main clinical manifestations of all the patients.

Variable	All cases	EES (%)	TCS (%)	p value
No. of cases	273	88 (32.2)	185 (67.8)	
Mean age (SD)	38.1 (12.9)	37.8 (13.8)	38.2 (12.3)	0.37
Male sex	128	41 (46.6)	87 (47.0)	1.00
Symptoms				
Headache	202	71 (80.7)	131 (70.8)	0.10
Impaired cognition	24	9 (10.2)	15 (8.1)	0.65
Visual deficits	134	47 (53.4)	87 (47.0)	0.37
Obesity	22	8 (9.1)	14 (7.6)	0.64
Hydrocephalus	39	11 (12.5)	28 (15.1)	0.71
Endocrine deficiencies	132	33 (37.5)	75 (40.5)	0.69

EES, endoscopic endonasal surgery; TCS, transcranial surgery.

### Tumor Characteristics and Extent of Resection

Pathological types, as well as tumor consistency, were similar between the two groups. The tumor characteristics and pathological types are listed in **Table 2**. There were no statistically significant differences noted. Among the 185 cases in the TCS group, GTR was achieved in 79 (89.8%) patients and subtotal resection in 9 (10.2%) patients. In the EES group, GTR was achieved in 143 (77.3%) patients; subtotal resection in 42 (22.7%) patients. The rate of GTR was statistically higher in the EES group, and the difference was statistically significant (p < 0.05).

**Table 2:** Tumor size, histopathological subtype, and consistency.

	All cases	EES (%)	TCS (%)	p value
No. of cases	273	88 (32.2)	185 (67.8)	
Mean tumor vol in cm^3^ (SD)	8.2 (7.9)	7.5 (8.4)	8.7 (7.1)	0.48
Tumor consistency				
Cystic	80 (29.3)	23 (26.1)	57 (30.8)	0.48
Solid	59 (21.6)	18 (20.5)	41 (22.1)	0.88
Mixed	134 (49.1)	47 (53.4)	87 (47.1)	0.37
Pathological type				
Adamantinomas	247 (90.5)	77 (87.5)	170 (91.9)	0.27
Papillary	26 (9.5)	11 (12.5)	15 (8.1)	0.27

EES, endoscopic endonasal surgery; TCS, transcranial surgery.

### Postoperative Complications

In this study, no surgery-related death occurred. Four (4.5%) of the 88 patients had postoperative leakage of CSF in the EES group and no patient in the TCS group. Two and 4 patients in the EES group and TCS group experienced meningitis, and no bacterial or fungal inflammations were found. All these differences were statistically significant with p < 0.05. Regarding other surgical complications, there were no significant differences in postoperative hemorrhage and seizures between the EES and TCS groups. **Table 3** demonstrates perioperative complications in the two groups.

**Table 3:** Main postoperative and perioperative complications.

Complications	All cases	EES (%)	TCS (%)	p value
No. of cases	273	88	185	
Hypopituitarism	173	47	126	0.02
Diabetes insipidus	179	45	134	<0.01
CSF leaks	4	4	0	0.01
Wound infection	10	2	8	0.51
Meningitis	6	2	4	1.00
Hemorrhage	5	2	3	1.00
Seizures	5	1	4	0.67
Death	0	0	0	1.00

CSF, cerebrospinal fluid; EES, endoscopic endonasal surgery; TCS, transcranial surgery.

### Visual Outcome

Most patients with preoperative vision and visual field loss experienced improvement after the operation. Among the 47 patients who had visual deficits preoperatively, 35 (74.5%) got visual improvement, and in the TCS group, 49 (56.3%) of 87 patients had improvement. The difference in remission rate was statistically significant (p < 0.05).

Two patients in the TCS group got blind in one eye. The remaining patients showed no change or slight deterioration in vision.

### Hypopituitarism and Diabetes Insipidus

Postoperatively, 47 (53.4%) and 126 (68.1%) patients presented hypopituitarism in the EES and TCS groups. Among patients with endocrine deficits preoperatively, endocrine function improved in 21 (63.6%) of 33 patients in the EES group and 23 (30.7%) of 75 patients in the TCS group. These data showed statistically significant differences.

Diabetes insipidus (DI) occurred in 45 (51.1%) patients and 134 (72.4%) patients in the EES group and TCS group, respectively, and the difference was statistically significant (p < 0.05).

### Tumor Recurrence

During the follow-up period, tumor recurrence occurred in 11 (12.5%) and 44 (23.8%) of the patients in the EES and TCS groups. The difference was significant (p < 0.05). The average time to recurrence was 8.3 months and 7.4 months in the two groups; no statistical difference was seen.

## Discussion

Craniopharyngioma is a tumor of low histological malignancy (WHO grade I) resulting from an anomaly of embryonic development (1). There are two clinicopathological subtypes (adamantinomas and papillary) with different characteristics, and the adamantinoma type (90%) is far more common than the papillary type (10%). Although craniopharyngioma is a benign tumor, it is among the most challenging brain tumors to manage regarding high rates of complications and recurrence. Surgery is the main method of treatment, and there remains controversy as to the optimal surgical treatment. Traditionally, craniopharyngiomas were operated on *via* a subfrontal, pterional, orbitofrontal, transcallosal, or transcortical approach. Recently, the endoscopic endonasal approach, wherein the tumor is resected transsphenoidal, has become more important during the past decade (14, 15). TCS and EES both have advantages and disadvantages. It should be noted that directly comparing TCS to EES is complicated regarding inherent selection bias (16, 17). Generally, the endoscopic endonasal approach is a better choice for intrasellar lesions and midline lesions. In contrast to the transcranial approach, the endoscopic approach can easily reach the sellar and parasellar regions, thus providing better close-up visualization of the optic nerve, optic chiasm, and pituitary stalk, and minimizes the retraction of the brain (18). Koutourousiou et al. (19) and Jane et al. (20) held the view that suprasellar craniopharyngiomas were better treated with craniotomy. Should craniopharyngioma extend too far laterally or posteriorly, the endonasal approach may not provide an entire view of the tumor, making maximal resection unlikely. Cavernous sinus or hypothalamic involvement may complicate the surgical resection and cause significant increases in mortality. In these suprasellar or intraventricular lesions, the extended endoscopic endonasal surgery (EEES) may be applied to better remove the sella turcica, the tuberculum sellae, and the posterior part of the planum sphenoidale (21). The combined use of endoscopic and microscopic may achieve better surgical effects through better visualization and protection of neurovascular structures.

Typically, the surgical outcome is closely associated with the extent of resection (3, 7, 22). Reports showed that the extent of resection is an independent predictor of tumor recurrence (7, 16, 17, 23–25). However, the close association of these tumors with critical neurovascular structures and locally aggressive characteristics make GTR difficult and lead to controversies surrounding the extent of resection in patients with craniopharyngiomas (10, 11, 26). Furthermore, radiosurgery has been proven to have the potential for better outcomes and decreasing mortality (3, 27–29). Subtotal resection surgery combined with radiotherapy has been advocated to protect hypothalamus–pituitary function and prevent tumor recurrence (23). In addition, neurosurgical expertise has an important impact on the extent of resection (24, 30–32), and tumor size may be a predictor of the postoperative functional outcome. Giant craniopharyngioma is associated with higher neurological, endocrinological, and hypothalamic morbidities postoperatively (33). In the present study, we prefer to achieve GTR if possible. The relationships between the tumor and the hypothalamus, pituitary, and optic chiasm were fully evaluated based on preoperative imaging, and an appropriate approach was chosen to allow adequate exposure of the tumor to the microscopic or endoscopic view. In some complicated cases, intraoperative ultrasound and MRI were applied to assess the extent of resection. Carai et al. (34) revealed that intraoperative ultrasound had a very good predictive value in neurosurgery to assist in intracerebral disease resection and improved the assessment ability of surgical resection (34, 35).

### Visual Outcomes

Visual impairment is the most common clinical manifestation affecting the quality of life of patients with craniopharyngioma. Approximately 62%–84% of patients present preoperative visual impairments (36). Endoscopic endonasal approach may have tremendous advantage in protecting the optic nerve and chiasm. In the current study, 74.5% of patients got visual improvement in the EES group, and in the TCS group, 56.3% of patients had improvement. Two patients in the TCS group got blind in one eye. The results were comparable to others. Some reports have found visual improvement rates reach 63% to 89% after endonasal resection while a lower rate of 25% to 53% after transcranial resection (17, 37).

The tumor often locates behind the optic nerve and optic chiasm, and it is inevitable to avoid retraction following the transcranial approach. In contrast, the endoscopic endonasal approach through the skull base can remove the tumor under direct close-up vision, which greatly reduces the retraction of optic nerves and chiasm (38). Qiao et al. (35) suggested that intraoperative visual evoked potential (VEP) can provide real-time warning for surgeons during the operation. In addition, optical coherence tomography (OCT) has become widely available and correlates well with the loss of visual function (39). It will be a more reliable outcome measurement compared to visual function testing and dilated fundoscopy in future studies.

### Cerebrospinal Fluid Leaks

CSF leakage remains one of the most common postoperative complications. Abrasion of the skull base and opening of the subarachnoid space make the transnasal approach more prone to CSF leakage than craniotomy. We routinely used autologous thigh broad fascia and vascularized flap to reconstruct the skull base in reducing postoperative leaks. In our study, CSF leaks occurred in 4.5% of patients in the EES group and none in the TCS group. Patients were then recovered through treatment of continuous lumbar drainage and antibiotics. The results are comparable to other studies that reported CSF leak rates of less than 10% (40, 41). A higher body mass index (BMI) and perioperative hydrocephalus may have an impact on the occurrence of CSF leakage (42).

### Postoperation Endocrine Deficits

Injury to the hypothalamic–pituitary axis, naturally, will cause endocrine deficits. Regardless of craniotomy or transnasal approach, the protection of hypothalamus and pituitary is the basis for GTR. In our study, DI occurred in 45 (51.1%) patients and 134 (72.4%) patients in the EES group and TCS group, respectively, consistent with other reports. The rate of endocrine deficits was reported to reach 52%–87% (2, 7, 27). During the operation, take care to identify and protect the superior hypophyseal arteries and pituitary stalk. Kawamata et al. (43) reported that preserving the pituitary stalk could reduce the risk of DI, nevertheless, increasing the risk of tumor recurrence. When dealing with craniopharyngiomas, sufficient preoperative discussion and preparation must be done, and treatment plans need to be individualized according to patient and tumor characteristics. Furthermore, a solid foundational knowledge of anatomy is imperative for decreasing the risks of surgery.

### Tumor Recurrence

There were statistical differences in the recurrence rates (12.5% EES vs. 23.8% TCS, p < 0.05) between the 2 groups in our study. It has been reported that the recurrence incidence was 0%–30% in cases of total resection (7, 10, 11, 44, 45). Komotar et al. (46) reported a recurrence rate of 18.4% and 28.2% in the endoscopic and transcranial group, with no statistical difference. Craniopharyngiomas characteristically tend to recur in patients with subtotal resection or partial resection. Patients received a second operation or radiotherapy when diagnosed with recurrence. Irradiation is considered efficient in preventing further growth or recurrence (27). Additionally, the calcified or cystic part may affect the effectiveness of radiotherapy. There are still concerns regarding radiation-induced toxicities and the potential risk of cyst enlargement that could cause severe compressive effects.

In summary, the endoscopic endonasal approach for resection of craniopharyngioma has a higher rate of total tumor resection and postoperative visual deficit recovery rate than the craniotomy approach. It is also better in terms of pituitary function protection, but the CSF leakage rate is slightly higher. Limitations to this study include selection bias and the development of surgical techniques. Due to the short follow-up period in this study, further study is needed in order to compare the therapeutic effects of the two surgical methods. With the development of neuroendoscopic technology and the accumulation of clinical experience of the surgeon, EES will be used more for the surgical treatment of craniopharyngioma.

## Conclusion

EES is associated with a superior visual outcome and lower rates of DI but has a higher risk for postoperative CSF leaks. These data support the view that EES is a safe and effective minimally invasive surgery, providing a viable alternative resection with less neurological injury and lower recurrence rates.

## Data Availability Statement

The raw data supporting the conclusions of this article will be made available by the authors without undue reservation.

## Ethics Statement

The studies involving human participants were reviewed and approved by the Ethics Committee of Wuhan Union Hospital. The patients/participants or their legal guardian/next of kin provided written informed consent to participate in this study.

## Author Contributions

CN: conceptualization, data curation, project administration, resources, formal analysis, software, visualization, and writing the original draft. YY: conceptualization, data curation, project administration, resources, formal analysis, software, visualization, and writing the original draft. JW: conceptualization, resources, investigation, and resources. HZ: supervision and writing—review and editing. XJ: conceptualization, investigation, resources, supervision, and writing—review and editing. HW: methodology, validation, data curation, writing—original draft, writing—review and editing, visualization, supervision, project administration, funding acquisition, resources, and data verification. All authors read and approved the final version of the article.

## Funding

This study has received funding from the National Natural Science Foundation of China (81974390).

## Conflict of Interest

The authors declare that the research was conducted in the absence of any commercial or financial relationships that could be construed as a potential conflict of interest.

## Publisher’s Note

All claims expressed in this article are solely those of the authors and do not necessarily represent those of their affiliated organizations, or those of the publisher, the editors and the reviewers. Any product that may be evaluated in this article, or claim that may be made by its manufacturer, is not guaranteed or endorsed by the publisher.
